# First-line endocrine treatment of breast cancer: aromatase inhibitor or antioestrogen?

**DOI:** 10.1038/sj.bjc.6601508

**Published:** 2004-01-06

**Authors:** Z-W Wong, M J Ellis

**Affiliations:** 1 National Cancer Centre, Singapore; 2 Breast Cancer Program, Washington University, 660 South Euelid, Campus Box 8056, St Louis, MO 63110, USA

**Keywords:** endocrine, breast cancer, aromatase inhibitor, antioestrogen

## Abstract

Until recently, endocrine therapy for breast cancer was relatively simple. If the tumour expressed hormone receptors, regardless of stage and age, tamoxifen was indicated. While this largely remains the case for premenopausal women, clinical trials in postmenopausal women have broadened our choice to include one of three selective aromatase inhibitors (AIs), the nonsteroidal agents anastrozole or letrozole and the steroidal agent exemestane. Comparative data concerning the efficacy, toxicity, tolerability and cost of AI *vs* tamoxifen continues to evolve with over 40 000 women slated to be involved in clinical trials. Currently, tamoxifen remains an appropriate choice for adjuvant treatment, and will remain so unless a clear survival advantage emerges for adjuvant AI therapy. However, anastrozole is widely seen as a useful alternative, with particular merit for patients prone to the development of serious tamoxifen side effects. For endocrine therapy naïve advanced disease, several trials have provided evidence that a nonsteroidal AI has replaced tamoxifen as optimal treatment. In the neoadjuvant setting, letrozole was also more effective than tamoxifen, both in terms of response rates and the incidence of breast-conserving surgery, and so AI therefore also dominates this evolving indication. The ongoing adjuvant clinical trials ask all the relevant questions regarding tamoxifen and AI in combination, sequence and duration, except for 5 years of an AI *vs* a longer period. For both the advanced and early-stage disease, resistance remains the key obstacle to overcome, and trials that combine endocrine agents with signal transduction inhibitors such as HER1 and HER2 kinase inhibitors, farnesyl transferase inhibitors, mTOR inhibitors as well as COX2 inhibitors are being developed in a concerted attempt to address this problem.

Formulating a view on the relative merits of aromatase inhibitors (AIs) and tamoxifen requires an examination of the available evidence regarding four major issues – efficacy, toxicity, tolerability/quality of life and cost. Each of these factors must be considered in the context of three major indications for endocrine treatment – neoadjuvant, adjuvant and advanced disease. While prevention is a fourth potential indication, there are no data yet from prevention trials to discuss. However, the arimidex, tamoxifen alone or in combination (ATAC) trial supports the hypothesis that AIs have chemopreventive properties, and clinical trials are now ongoing. For this review, ‘antioestrogen’ is synonymous with tamoxifen, because the two other approved antioestrogens either have no comparative data with AIs (toremifene) or published comparisons with AIs were not conducted in the first-line setting (fulvestrant).

## 

### What is first-line endocrine treatment for breast cancer?

First-line treatment for breast cancer is the easiest to define in terms of a new diagnosis, whether a new primary or initial systemic relapse. However, these events do not necessarily equate to first-line endocrine therapy because the patient may have already been exposed to raloxifene for osteoporosis or tamoxifen for chemoprevention. There are unfortunately only limited data to guide the clinical management of these patients. The recommendation to use an adjuvant AI in selective oestrogen receptor modulation (SERM)-exposed patients (Winer *et al*, 2002) stems from advanced disease trials in which patients either relapsing on or after tamoxifen therapy still have a reasonable chance of responding to AIs (Smith and Dowsett, 2003). Similarly, a patient with newly diagnosed hormone receptor-positive advanced disease is highly likely to have already been treated with adjuvant tamoxifen. If the relapse occurred on tamoxifen or within 6 months to 1 year of stopping tamoxifen, then the patient is considered to be in a ‘second-line endocrine therapy’ category, and the standard recommendation is an AI (Smith and Dowsett, 2003). Patients who relapse after an interval of more than 1 year are traditionally considered ‘first-line’ endocrine therapy candidates, as the standard of care was to reintroduce tamoxifen therapy. However, a recent large study demonstrated that this is a relatively ineffective strategy, with an odds ratio favouring letrozole treatment approaching 4 in this subgroup (Mouridsen *et al*, 2003). Perhaps, any patient who has completed a course of adjuvant tamoxifen could really be considered to be a candidate for second-line endocrine therapy upon relapse, regardless of how long ago the exposure to tamoxifen was.

### Antioestrogens and AIs have distinct mechanisms of action

Tamoxifen (antioestrogen) and aromatase inhibition (oestrogen deprivation) are distinct approaches to oestrogen-dependent breast cancer and efficacy, tolerability and toxicity differences stem from these pharmacological fundamentals (Smith and Dowsett, 2003). Unlike tamoxifen, AIs do not exhibit intrinsic hormonal properties but affect oestrogen receptor (ER) function indirectly by blocking the conversion of adrenal androgens to oestrogen in the peripheral (i.e. nonovarian) tissues of postmenopausal women, including the breast itself (Miller, 1991). When deprived of oestrogens, ERs cannot bind to DNA, and are therefore incapable of direct involvement in signalling. As a consequence, the side effects of AIs stem from oestrogen deprivation – for example, bone loss and atrophic vaginitis. In contrast, tamoxifen has an intrinsic endocrine action by binding to ERs with high affinity and activating ER dimerisation and DNA binding. Tamoxifen-bound ER has altered gene-regulatory properties referred to as SERM. In several normal tissues, tamoxifen-bound ER is active and promotes, for example, bone mineralisation and endometrial proliferation. In these instances, tamoxifen is acting as an ‘oestrogen mimic’ or receptor agonist. As a consequence, the serious side effects of tamoxifen are remarkably similar to oestrogen replacement therapy (ERT) – venous thrombosis, pulmonary embolus, stroke and endometrial cancer (ATAC, 2002). Like ERT, maintenance of bone mineral density is an advantage of tamoxifen. These agonist effects may, however, limit efficacy. Aromatase inhibitors do not interact with ER; so potential resistance mechanisms related to SERM agonist effects on tumour cells are avoided. Preclinical models with MCF7 xenografts engineered to overexpress aromatase (Brodie *et al*, 2003), as well as models of tamoxifen-dependent tumour growth (Gottardis and Jordan, 1988), support this explanation for the therapeutic advantage of AIs.

### First-line therapy for advanced disease

This discussion will focus on anastrozole, letrozole and exemestane. Fadrozole, a second-generation nonsteroidal AI, has been shown to be equivalent to tamoxifen (Thurlimann *et al*, 1996) but inferior to letrozole (Tominaga *et al*, 2003) in advanced breast cancer, and will not be discussed further, as drug approval is restricted to Japan.

### Anastrozole

Anastrozole has been compared with tamoxifen as first-line endocrine therapy in more than 1000 postmenopausal metastatic breast cancer patients with either positive or unknown hormone receptor status in two randomised double-blind trials. The study from Europe (Bonneterre *et al*, 2000) showed similar time to progression (TTP) and clinical benefit rates (defined as CR+PR+disease stabilisation of >24 weeks) in the anastrozole and tamoxifen arms of the study (8.2 *vs* 8.3 months and 56.2 and 55.5%, respectively). In the North American study (Nabholtz *et al*, 2000), anastrozole was significantly superior to tamoxifen for both TTP and clinical benefit rates (11.1 *vs* 5.6 months, *P*=0.005 and 59.1 *vs* 45.6%, *P*=0.0098, respectively). It is important to note that there was a marked difference in the prevalence of known receptor-positive patients between the two studies (45 and 89%, respectively), potentially accounting for the differences observed. The combined data analysis from the two studies showed anastrozole to be equivalent to tamoxifen in TTP at a median follow-up of 18.2 months (Bonneterre *et al*, 2001), but, in a retrospective subgroup analysis, anastrozole was superior to tamoxifen in the subgroup with known receptor-positive tumours with respect to TTP (10.7 *vs* 6.4 months). In addition, although anastrozole had similar objective response rates (29.0 *vs* 27.1%), the clinical benefit rates were higher (57.1 *vs* 52.0%). It is therefore reasonable to conclude that anastrozole is superior in terms of TTP for patients with receptor-positive tumours. The controversy over the interpretation of unplanned retrospective analyses used by these investigators underscores the inappropriateness of including patients with unknown receptor status in these studies. To avoid this problem, patients without hormone receptor data are likely to be excluded from future endocrine therapy trials.

### Letrozole

Letrozole was compared with tamoxifen in a randomised trial of 907 patients, whose tumours were receptor positive or unknown (Mouridsen *et al*, 2001). Recurrence during adjuvant antioestrogen therapy or within the following 12 months or prior endocrine therapy for advanced disease precluded enrollment, and only one prior chemotherapy regimen for metastatic disease was allowed. Letrozole treatment was found to be superior to tamoxifen, with a median TTP of 41 weeks *vs* 26 weeks. The median time to treatment failure, overall response rate and clinical benefit rate were significantly better in favour of letrozole (40 *vs* 25 weeks; 30 *vs* 20%, *P*=0.0006 and 49 *vs* 38%, *P*=0.001). At a recent update after a median of 32 months of follow-up, the superiority of letrozole over tamoxifen in the TTP (median, 9.4 *vs* 6.0 months, respectively; *P*<0.0001), time to treatment failure (median, 9 *vs* 5.7 months, respectively; *P*<0.0001), overall objective response rate (32 *vs* 21%, respectively; *P*=0.0002) and overall clinical benefit were confirmed (Mouridsen *et al*, 2003). Interestingly, the total duration of endocrine therapy (‘time to chemotherapy’) was significantly longer for first-line letrozole than for first-line tamoxifen (median, 16 months *vs* 9 months, *P*=0.005). Similarly, the time to worsening of Karnofsky performance score was also significantly delayed with letrozole compared with tamoxifen, underscoring the palliative benefits of a first-line AI approach.

### Exemestane

Exemestane has been compared with tamoxifen in 117 previously untreated breast cancers with either positive or unknown hormone receptors in a randomised phase 2 EORTC study (Dirix *et al*, 2001). The overall response rate was better for exemestane than tamoxifen (44.6 *vs* 14.3%). Time to progression has not been analysed, as this is the primary end point of a Phase 3 extension of this ongoing study.

### Interpretation of the advanced disease data

The letrozole experience, by virtue of the consistent findings and highly significant effects on the TTP and response rate, provided the best evidence for the advantage of first-line AI treatment. These advantages were independent of a number of baseline covariates, including the site of disease, prior use of tamoxifen and known or unknown hormone receptor status (Mouridsen *et al*, 2003). The overall survival data from this trial have raised some interesting issues regarding the sequencing of tamoxifen and letrozole. Whereas women on letrozole had a clear trend towards improved survival during the first 2 years, this survival benefit was ‘lost’ after further follow-up (median survival 34 months with letrozole *vs* 30 months for tamoxifen). As a result, several parametric tests were not significant when applied to the data (*P*=0.53 log rank, *P*=0.079 Wilcoxon). However, the nonparametric Kolmogorov–Smirnov-type test did indicate that the two survival curves were significantly different in favour of letrozole between 6 and 20 months (*P*=0.003). An obvious explanation for the apparently ‘temporary’ survival advantage for first-line letrozole is that cross-over to letrozole also improved survival as second-line treatment on the tamoxifen arm. The alternative hypothesis is that cross-over to tamoxifen decreased the survival rate of patients who received letrozole first. This possibility is not completely far-fetched. Prolonged oestrogen deprivation causes oestrogen hypersensitivity (Santen *et al*, 2003), which might also prime a cell to perceive tamoxifen as an agonist. Clearly, trials that investigate the endocrine management of patients with AI-resistant advanced disease are of major priority. The options now include tamoxifen, fulvestrant (Howell, 2000) or switching from a nonsteroidal AI to exemestane (Lonning *et al*, 2000). The data available on these strategies are limited, but the response rates are generally less than 10%. In fact, perhaps the most active approach may be pharmacological doses of oestrogen (Ingle, 2002), which would concur with the apoptotic response of long-term oestrogen deprived cells to the reintroduction of oestrogen (Song *et al*, 2001).

From the view of current practice, the higher response rate and lower incidence of early progression when treating with letrozole first translate into a quality of life benefit and staves off, the moment palliative chemotherapy becomes necessary. This efficacy distinction between AIs and tamoxifen is perhaps most important for patients with disease involving vital organs, where disease progression is most likely to trigger the use of palliative chemotherapy. Aromatase inhibitors are quite active against visceral disease and superior to tamoxifen in this regard (Mouridsen *et al*, 2003). For patients with low volume disease and low symptom burden, the order of treatment is arguably less critical, because several endocrine agents are likely to be used in the sequence, regardless of treatment response.

### Aromatase inhibitor *vs* tamoxifen as adjuvant therapy for breast cancer

Around 40 000 patients are either scheduled to enroll or have enrolled in adjuvant studies involving AIs, but the only study that has been reported so far is the ATAC trial (ATAC, 2002). In all, 9366 patients were recruited, of whom 3125 were randomly assigned anastrozole, 3116 tamoxifen and 3125 a combination of both. A total of 7839 patients (84%) were known to be receptor-positive. With a median follow-up of 33.3 months, the disease-free survivals (defined as freedom from local recurrence, systemic recurrence, contralateral breast cancer or death as first event) at 3 years were 89.4% on anastrozole and 87.4% on tamoxifen (hazard ratio 0.83 (95% CI 0.71–0.96), *P*=0.013). Results with the combination were not significantly different from those with tamoxifen alone (87.2%, 1.02 (0.89–1.18), *P*=0.8). Improvements in disease-free survival with anastrozole were only seen in the receptor-positive population. The incidence of contralateral breast cancer was also significantly lower with anastrozole than with tamoxifen (odds ratio 0.42 (0.22–0.79), *P*=0.007). After 47 months of follow-up and 1373 events, the DFS estimates were 86.9 and 84.5% for anastrozole and tamoxifen, respectively (Buzdar, 2002). The actual events that make up these statistics are provided in Table 1

**Table 1:** Updated efficacy results of ATAC trial with a median follow-up of 47 months

	**AN (*n*=3125)**	**TAM (*n*=3116)**	**AN+TAM (*n*=3125)**
Deaths before recurrence	108	109	100
First event	413	472	488
Locoregional	84	101	107
Distant	195	222	246
CL (invasive)	20	35	30
CL (DCIS)	5	5	5

AN=anastrozole; TAM=tamoxifen; CL=contralateral breast cancer; DCIS=ductal carcinoma *in situ*.

. The difference in DFS estimates was slightly greater in the hormone-receptor positive subgroup (89.0 *vs* 86.1%). These differences remain modest in absolute terms and persistent divergence of the curves with further follow-up, as well as supportive data from other trials, will be very reassuring to patients embarking on anastrozole as adjuvant treatment. With each trial update, the risks and benefits will be weighed (Figure 1

**Figure 1:**
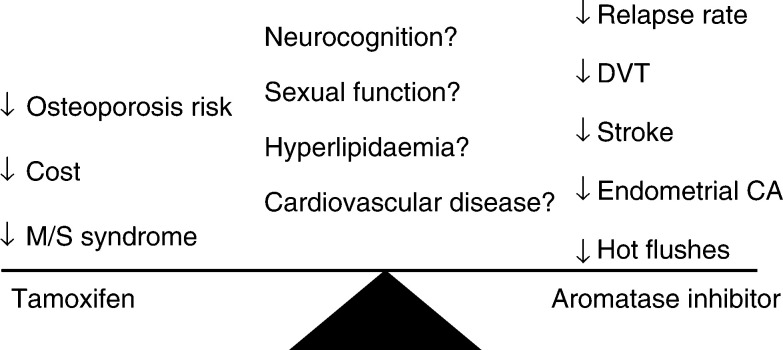
Comparison of toxicities of AI *vs* tamoxifen from the ATAC trial (CA=cancer, DVT=deep venous thrombosis, M/S=musculoskeletal).

). Only when the efficacy data become stronger, particularly with respect to overall survival, or the differences in toxicity becomes greater, are we likely to see a change in the ASCO technical review on adjuvant AIs, which emphasises the strength of the tamoxifen experience and the preliminary nature of the ATAC trial data (Winer *et al*, 2002, 2003). This view was also endorsed by the recent St Gallen Consensus Panel, which also recommended that anastrozole be used only in postmenopausal women in whom tamoxifen is contraindicated, or is not tolerated (Goldhirsch *et al*, 2003).

### Neoadjuvant AI therapy

The results of a randomised comparison of letrozole and tamoxifen as neoadjuvant therapy for postmenopausal women with Stage 2 and 3, hormone receptor-positive disease are noteworthy from a number of standpoints (Eiermann *et al*, 2001). Letrozole produced a higher clinical and radiological response rate than tamoxifen, and also the incidence of breast-conserving surgery was superior (all patients in the trial were considered ineligible for breast-conserving surgery at baseline). This result therefore mirrors the conclusion from the ATAC trial that a third-generation AI is more effective than tamoxifen as treatment for primary breast cancer. If neoadjuvant and adjuvant endocrine outcomes continue to be concordant in this way, future novel endocrine strategies may well use the neoadjuvant setting to establish essential preliminary data to support a definitive large-scale adjuvant trial. Another noteworthy aspect of the study concerned the opportunity to conduct biomarker research to better understand the molecular basis for the therapeutic response to endocrine agents. For example, an analysis of trial outcomes according to HER1 (EGFR) and HER2 (ErbB2) status demonstrated that the difference in the effectiveness of letrozole and tamoxifen was particularly marked for tumours that coexpressed ER and HER1 and/or HER2 (Ellis *et al*, 2001). This finding has led to an effort to examine the outcome of the ATAC study according to HER2 expression status (Dowsett M, personal communication). Further biomarker research has also shown that the cell cycle-related biomarker Ki67 is suppressed to a greater extent by letrozole than tamoxifen, regardless of HER1 and HER2 expression status. This would suggest that part of the superior efficacy associated with aromatase inhibition is related to a greater inhibition of tumour proliferation (Ellis *et al*, 2003). A significant obstacle to the routine adoption of neoadjuvant endocrine therapy is the absence of a large validation study in which the outcomes of patients who received neoadjuvant AIs are directly compared with a group treated conventionally (either with neoadjuvant chemotherapy or immediate surgery). Conducting a trial of this nature is a priority, given the potential of research in this clinical context, as well as the inherent advantages from a wider acceptance of a low-toxicity approach to neoadjuvant treatment for older patients (Wong and Ellis, 2003).

### Toxicity and tolerability

Differences in the side effect profile between tamoxifen and anastrozole are marked, and arguably more likely to influence the current prescribing practice than differences in efficacy. There is no difference between anastrozole and tamoxifen in terms of cataracts, ischaemic cardiovascular events, fatigue or asthenia, mood disturbances and nausea and vomiting. However, anastrozole was associated with fewer endometrial cancers, vaginal bleeding and discharge, cerebrovascular events, venous thromboembolic events and hot flushes. Tamoxifen caused fewer musculoskeletal disorders and fractures. These data have led the ASCO technical panel and the St Gallen Consensus panel to emphasise that anastrozole might have an important role for those at particular risk for severe tamoxifen side effects. On the other hand, anastrozole should be prescribed with caution in patients with osteopenia/osteoporosis. The increase in fracture risk associated with anastrozole was evident even at the first analysis, suggesting that the decline in bone strength may occur very rapidly in some patients, perhaps even before major changes in bone mineral density are detected. Arguably, all patients on adjuvant AI therapy should receive vitamin D and calcium supplements, be encouraged to exercise, and those with evidence for bone loss at baseline should also receive an oral bisphosphonate, until practice guidelines are established. The ASCO technical review and the St Gallen Consensus Panel continue to emphasise the lack of safety data regarding long-term oestrogen deprivation. For example, AIs have been reported to alter cholesterol and lipoprotein metabolism with potentially deleterious effects on cardiovascular health (Elisaf *et al*, 2001). There are also concerns about the effects of long-term oestrogen deprivation on memory and risk for dementia. Regions of the brain such as the hippocampus and amygdala involved in learning and memory are rich in ER, as oestrogen regulates synapse formation and induces choline transferase and acetylcholinesterase, both critical to memory function (McEwen *et al*, 1997). While tamoxifen has been implicated in decline in cognitive function (Paganini-Hill and Clark, 2000), these data do not come from double-blind randomised placebo-controlled trials, and so the issue remains controversial. The positive influence of oestrogen on the general health, vitality, mental health, depressive symptoms, or sexual satisfaction has recently been seriously questioned (Rossouw *et al*, 2002; Hays *et al*, 2003), and so, while these issues need careful study, the potential of AIs to be unduly toxic in the long run should not be overstated.

### Quality of life

For metastatic breast cancer, balancing the toxicity-to-benefit ratio of any treatment is vital in maintaining the quality of life in otherwise incurable patients with a limited lifespan. In more than 900 patients enrolled in a multi-centre trial evaluating the efficacy of letrozole compared to tamoxifen as first-line therapy in metastatic breast cancer, letrozole offered a significantly longer quality-adjusted survival than tamoxifen (mean duration for TWiST or time without disease progression or toxicity was 10.1 months compared to 7.6 months for tamoxifen, *P*=0.0004) (Irish *et al*, 2002). Some have suggested that letrozole may be superior to anastrozole in terms of gastrointestinal side effects, nausea and hot flushes (Makris *et al*, 2002).

A European study that evaluated more than 500 women with early breast cancer after 2–3 years of adjuvant tamoxifen found that the adverse effects of tamoxifen therapy on quality of life may be under-reported (Coombes *et al*, 2003). Using the FACT-ES (Functional Assessment of Cancer Therapy or FACT-B, plus ES, an 18-item five-point endocrine subscale), eight symptoms that were most problematic for >10% of the patients included: hot flushes, night sweats, vaginal dryness, loss of interest in sex, weight gain, bloatedness, breast sensitivity and mood swings (Fallowfield *et al*, 1999). However, results from the NSABP-P1 study showed that there was no difference between tamoxifen and the placebo arms with regard to depression, overall physical or mental quality of life and weight gain, though those on the tamoxifen arm have more vasomotor (hot flushes) and gynecological symptoms (vaginal discharge) and difficulties in sexual functioning (Day, 2001; Day *et al*, 2001). The final analysis of the effects of AIs on the quality of life is still pending, but there are preliminary evidences that anastrozole may cause a decline in sexual functioning compared to tamoxifen (Fallowfield *et al*, 2002). While the ATAC trial showed that postmenopausal symptoms such as hot flushes are fewer with anastrozole, this is arguably offset by the higher prevalence of musculoskeletal complaints, which may be quite marked in some patients (Sainsbury, 2002).

### Cost

Aromatase inhibitors cost several fold more than tamoxifen, a critical issue for seniors without a prescription drug benefit or in the context of government-run health-care systems. From a pharmacoeconomics perspective, using a decision model (Markov process) based on the UK National Health Service, letrozole is a cost-effective alternative first-line therapy, compared with tamoxifen for postmenopausal women with advanced breast cancer, achieving additional life-years at a mean incremental cost per life-year gain of £2342 (Karnon and Jones, 2003). In another cost-effectiveness analysis undertaken in Canada using a decision model, quality-adjusted progression-free survivals between letrozole and anastrozole were comparable. Letrozole and anastrozole cost Can$2883 and Can$2847 per patient, respectively, which were marginally higher than tamoxifen at Can$ 2258 per patient. This translated into an incremental cost over tamoxifen per quality-adjusted progression-free year of $12,500 for letrozole and $19 600 for anastrozole (Dranitsaris *et al*, 2003).

### Conclusion and future directions

The third-generation AIs are a welcome additional option for the endocrine therapy of hormone receptor-positive breast cancer in postmenopausal women. While nonsteroidal AIs are more effective than tamoxifen for the treatment of advanced disease, the absolute reduction in relapse-free survival for adjuvant AIs over tamoxifen is modest, and the results of several other large adjuvant trials are currently pending. Since there is currently no evidence that the use of an AI increases the number of patients cured of their disease, we consider the impact of AI therapy to be relatively modest, at least for the moment. The efficacy differences between tamoxifen and an AI are likely to remain fairly narrow, because ultimately these agents target the same signal transduction pathway. A critical focus is, therefore, to develop and translate insights into the molecular basis for endocrine therapy resistance. Neoadjuvant studies are proving part of the key, since tissue is readily available for genomic and proteomic approaches. Efforts should now also be made to profile samples from advanced disease, in order to fully understand the nature of the problem, since acquired resistance is not adequately addressed in neoadjuvant studies. Combining AIs and antioestrogens with a second signal transduction inhibitor to prevent or even reverse endocrine therapy resistance is now under investigation. The number of studies is rapidly expanding, and involve a spectrum of agents including COX2 inhibitors (Harris *et al*, 2000), HER1 and HER2 kinase inhibitors (Kurokawa and Arteaga, 2001), farnesyl transferase inhibitors (Johnston *et al*, 2003) and mTOR inhibitors (Mita *et al*, 2003). Combining endocrine therapy with the rational use of these agents may be our best hope for more dramatic advances in the treatment of this common disease.
